# ATP2A2 rs3026468 does not associate with quadriceps contractile properties and acute muscle potentiation in humans

**DOI:** 10.1152/physiolgenomics.00085.2018

**Published:** 2018-12-07

**Authors:** Eric A. Kirk, Shiva M. Singh, Charles L. Rice

**Affiliations:** ^1^School of Kinesiology, Faculty of Health Sciences, the University of Western Ontario, London, Canada; ^2^Department of Biology, Molecular Genetics Unit, The University of Western Ontario, London, Canada; ^3^Department of Anatomy and Cell Biology, Schulich School of Medicine and Dentistry, The University of Western Ontario, London, Canada

**Keywords:** ATP2A2, muscle potentiation, rs3026468, skeletal muscle, voluntary strength

## Abstract

The ATP2A2 gene encodes the SERCA protein required for active calcium reuptake to the sarcoplasmic reticulum in cardiac and slow-twitch skeletal muscle. The ATP2A2 rs3026468 variant has been associated with voluntary strength phenotypes in humans but requires further validation. Here we investigated a homogenous cohort of 80 young, healthy, active Caucasian males who were assessed for maximal isometric strength, voluntary activation, stimulated contractile properties, and muscle potentiation in the quadriceps. A dynamometer was used to record knee extensions, and electrical stimulation was applied to the thigh to elicit a twitch response. DNA was isolated from cheek swabs, and the rs3026468 genotypes were assessed by TaqMan primer quantitative PCR. The results show no association between ATP2A2 rs3026468 variants and muscle strength measures. We conclude there is no effect of the rs3026468 variant in our cohort and that functional influences do not likely contribute to contractile property differences in young healthy men.

## BACKGROUND/MOTIVATION FOR THE STUDY

Few investigations have performed comprehensive physiological analysis to validate single nucleotide polymorphisms (SNPs) associated with skeletal muscle strength in humans. A prior investigation used a fine-mapping approach of 122 SNPs within a skeletal muscle strength quantitative trait locus on 12q22-23 (5) and identified rs3026468 as a candidate linked SNP. The rs3026468 SNP is located within the ATPase sarcoplasmic/endoplasmic reticulum Ca^2+^ transporting 2 (ATP2A2) gene. This gene is importantly expressed in cardiac and type II skeletal muscle encoding the SERCA2 protein required for active calcium reuptake to the sarcoplasmic reticulum necessary for the contraction-relaxation cycle. In rodent models, mutations to the orthologous ATP2A2 gene result in altered Ca^2+^ handling and functional changes to cardiac muscle contraction and relaxation. In humans, mutations to ATP2A2 result in the loss of adhesion between epithelial cells causing Darier disease (OMIM124200), a dominantly inherited skin disorder, and in the adductor pollicis muscle there is prolonged one-half relaxation time (1). If the ATP2A2 rs3026468 variant does influence voluntary strength during isometric or dynamic contractions via Ca^2+^ handling during the contraction-relaxation cycle (5), we hypothesize there would likely be functional differences detected in electrically stimulated contractile properties and muscle potentiation. We investigated the involvement of the rs3026468 variant in voluntary strength, voluntary activation, stimulated contractile properties, and muscle potentiation in our cohort.

## PHENOTYPE

Participants were secured in a knee extension dynamometer (Cybex Humac Norm, CSMi Medical Solutions). Maximal voluntary isometric contractions (MVC) were ~5 s in duration, with 5 min rest between attempts until peak torque did not differ by 5% or less (Power 1401, Spike2, version 7.7, Cambridge Electronic Design). Electrical stimulation was delivered through aluminum electrodes positioned on the anterior thigh at distal and proximal locations (3). The current of square-wave pulses (100 µs, 400 V) was increased to elicit maximal twitch torque responses (DS7AH, Digitimer). The rate of torque development (RTD) and one-half relaxation time (HRT) were calculated from the twitch (3). Voluntary activation (VA) was assessed by the interpolated twitch technique (4). Muscle potentiation was measured as the ratio of the stimulated twitch torque amplitude 1 s after MVC to the resting twitch torque amplitude before MVC.

### 

#### Cohort details.

In a retrospective study design based upon findings from Windelinckx et al. (2011) (5), 80 nonrelated Caucasian adult males (19–31 yr) were recruited and gave informed written consent with approval from the local University ethics board. All participants were recreationally exercised and from the local University population. Exclusion criteria included: overly sedentary; systematically trained; known neuromuscular, respiratory, cardiovascular, or metabolic illness; caffeine consumption 3 h before testing; and intensive exercise 48 h before testing.

#### Type of study.

Candidate SNP.

#### Details of the SNP studied.

Single SNP accession number: rs3026468. Gene name: ATPase sarcoplasmic reticulum Ca^2+^ transporting 2 (ATP2A2). SNP detection: DNA was isolated from human cheek swabs (QIAamp DNA blood mini kit) and assessed on a NanoDrop 2000c. Each 20 µl reaction consisted of: 10 µl master mix (Luna Universal Probe qPCR master mix, New England Biolabs), 0.5 µl TaqMan probe (rs3026468, ThermoFisher Scientific), 10–20 ng of template DNA, and water. Real-time quantitative PCR was performed on a CFX Connect real-time PCR detection system (Bio-Rad), and genotypes were determined by end point fluorescence of VIC and FAM signals using CFX manager software, version 3.1 (Bio-Rad). Heterozygous DNA template controls (301 nt in length) were synthesized (ThermoFisher Scientific) to contain either the common (T) or rare (A) allele for the rs3026468 variant region and acted as positive controls (Supplemental Table S2). (The online version of this article contains supplemental material.)

#### Analysis model.

The genotype distribution was tested for Hardy-Weinberg equilibrium (HWE) using an open access software package (http://www.oege.org/software/hwe-mr-calc.shtml) (2). Statistical analysis between genotype groups was performed in R (version 3.4.3). Each phenotype (MVC, voluntary activation, RTD, HRT, and potentiation) was grouped by their respective genotype, and the Shapiro-Wilk normality test determined the distribution was nonparametric. Genotype groups were compared by the Wilcoxon rank sum test with the alpha set to 0.05; if significance was found, the Bonferroni correction was used. Post hoc power calculations were made based on presented knee extension data from Windelinckx et al. 2011 (5) (power 0.80, alpha 0.05), determining our cohort was sufficient in size to detect differences.

## RESULTS

The genotype distribution was in HWE (χ^2^ = 0.99, *P* > 0.05) with 16 heterozygotes in our cohort (*n* = 80), with no individuals homozygous for the minor allele (frequency 0.1). Anthropometric characteristics and phenotypes of skeletal muscle strength, voluntary activation, and stimulated contractile properties are presented in Supplemental Table S1. There were no differences in voluntary MVC, voluntary activation, RTD, HRT, and potentiation between genotype groups for the ATP2A2 rs3026468 variant (GRCh38.p7, Chr 12, Fwd 110,320,276).

## INTERPRETATION

The ATP2A2 rs3026468 variant (5) does not appear to influence interindividual strength differences or stimulated contractile properties in the human quadriceps muscle. If functionally important for strength, differences in Ca^2+^ handling by altered SERCA2 efficacy should have been detected during our measures of RTD, HRT, and muscle potentiation. We observed an allelic distribution in HWE, with the minor allele in agreement with values from the 1000 Genomes and TOPMED projects. Limitations include small sample size and that only quadriceps muscles were assessed. In summary, in healthy muscle of young men, voluntary strength and associated properties are not likely explained by the rs3026468 variant.

## GRANTS

This work is supported by the Natural Sciences and Engineering Research Council of Canada discovery grant.

## DISCLOSURES

No conflicts of interest, financial or otherwise, are declared by the authors.

## AUTHOR CONTRIBUTIONS

E.A.K., S.M.S., and C.L.R. performed experiments; E.A.K., S.M.S., and C.L.R. analyzed data; E.A.K., S.M.S., and C.L.R. interpreted results of experiments; E.A.K., S.M.S., and C.L.R. prepared figures; E.A.K., S.M.S., and C.L.R. drafted manuscript; E.A.K., S.M.S., and C.L.R. edited and revised manuscript; E.A.K., S.M.S., and C.L.R. approved final version of manuscript.
